# Application of Local Approaches to Crack Development in Structural Materials

**DOI:** 10.3390/ma19153157

**Published:** 2026-07-23

**Authors:** Vladislav Kozák, Jiří Vala

**Affiliations:** Institute of Mathematics and Descriptive Geometry, Faculty of Civil Engineering, Brno University of Technology, 602 00 Brno, Czech Republic; vladislav.kozak@vut.cz

**Keywords:** structural materials, crack initiation and development, computational modelling, extended finite element method (XFEM), 62.20.mt, 46.50.+a, 02.70.Dh, 02.70.Bf

## Abstract

The scientific community and engineers are interested in simulating and analysing the behaviour of individual components and complex structures. This review article highlights progress in the area of modelling of structural materials based on the use of the finite element method. In addition to being described deterministically, the situation ahead of a possible stress concentrator is also defined using modern statistical techniques, and the behaviour simulation is explained in terms of length scales. Crack development and generation in the component are the main uses of the description of the impact of local microstructure on macrostructure. There are two predominant types of multiscale analysis: hierarchical and concurrent. Hybrid types also exist, but these are beyond the scope of this paper. Mainly, two-scale hierarchic simulations are illustrated hereafter. The modelling is dedicated to the following groups of materials: (i) crack initiation around inclusions, carbides at grain boundaries, and natural or artificial stress concentrators; (ii) composites with short or long fibres and pronounced interfaces; and (iii) combinations of (i) and (ii) in the case where the grain structure behaves like a short fibre. The authors draw on many years of experience in the field of numerical methods and, in particular, the modified finite element method.

## 1. Introduction

For the safety of structural components, it must be demonstrated that no crack initiation or even propagation will occur. Environmental effects must be included for a safe description. Various combinations of mechanical, thermal, and external stress conditions can result in brittle fracture. The following crucial issues must be considered from a fracture mechanics perspective, determining whether a material satisfies the requirements for resistance to catastrophic failure: (i) the transferability of fracture toughness data determined on small specimens to a component of much larger size, and (ii) the prediction with an acceptable probability of brittle fracture.

Partial differential equations, supplied by some initial and boundary conditions, are frequently used to model physical systems. Only certain geometries have access to the precise answer or a closed form of the analytical solution. In more generic situations, the exact solution can be approximated using numerical techniques. A numerical simulation’s outcome is rarely precise. Engineering-related fields have produced numerical simulations as an industrial output. A common issue is comparing simulations with experiments to improve the simulation process and yield better results. It appears that engineers and mathematicians have created comparable techniques related to the finite element method (FEM). W. Ritz and B. G. Galerkin [1,2] employed variational techniques to solve partial differential equations at the beginning of the 20th century, and five decades later came the first FEM computations [3], followed by the gradual development of proper mathematical theory [4].

Many distinct theories have been put forth in the past century and even earlier to study elasticity, plasticity, fracture, and other related phenomena. As a result, their mathematical and computational unifications are difficult or impossible in many engineering applications where there is no reasonable physically motivated linearisation (such as the principles of proportionality and additive superposition in computational mechanics). Scientists were able to connect disparate length scales and develop theories ranging from the subatomic to the macroscopic level thanks to advances in solid-phase physics, numerical mathematics, computer architecture, and parallel systems. For example, the realisation that the motion and accumulation of defects (e.g., dislocations) is the microscopic process that causes plasticity encouraged the creation of a theoretical tool. A multiscale geometry that captures the contributions of both smooth geometrical changes at the macroscopic level and non-smooth geometrical changes at sub-macroscopic levels—called disarrangements, encoding the presence of cracks and defects in the continuum—is provided by structured deformation [5], which was introduced in theoretical mechanics, generalizing the kinematics of elastoplastic behaviour of deformable bodies by introducing multiplicative decomposition of finite strain tensors [6,7,8,9,10].

For the analysis of fractured zones, the approach described in [11], revising the classical Mazars model [12,13], is available. Its ingredients may include the introduction of a required ad hoc damage factor [14], its anisotropic generalization (see [15]), the smoothing gradient damage technique [16], an alternative stochastic approach cf. [17], or the exploitation of fractal dimensions for quantification of crack distributions [18]. Such an approach needs some energy regularization [19,20]. Consequently, nonlocal formulae can also be utilised for the evaluation of the possible initiation of a system of macroscopic cracks, whose behaviour can be studied on basis of the cohesive model [21,22,23]; its effective implementation can be supported by the extended finite element method (XFEM) [24,25,26,27,28] or some similar approach from the wider class of partition of unity methods [29].

The stress singularity at the crack tip is usually represented by singular elements in traditional FEM [30]. By adding asymptotic crack-tip enrichment functions, XFEM can manage crack-tip singularities. However, it should be mentioned that asymptotic crack-tip enrichment functions are not always required in methods that combine XFEM with cohesive models [31]. Additionally, as the crack progresses, FEM necessitates remeshing at each level, increasing the computational needs; in contrast, XFEM does not require remeshing or needs little local remeshing only. Since mesh is independent of crack position, monitoring the time history of points in FEM is complicated; however, in XFEM, this is not a problem. Frequent exploitation of XFEM in the last several decades can be illustrated by the detailed classification of classical, modified, and improved XFEM approaches along with other concurrent computational techniques in [32], which contains 194 references.

Advanced materials with complicated responses under stress, such as composites, piezoelectric materials, and functionally graded materials, have been easier to research because of XFEM. Because XFEM can simulate crack propagation without the need for remeshing, it is a crucial tool for forecasting how these materials will behave under different circumstances, which helps with the creation of more robust and effective materials. The capacity of XFEM to precisely simulate micro-scale and nano-scale fracture behaviours will be crucial as the field of materials science advances toward the creation of smart materials and nanotechnology. This ability will aid in the creation of next-generation materials, which will be essential for upcoming technologies like biocompatible materials and flexible electronic devices.

With an emphasis on the notable advancements in research over the past few years that have not been covered by well-organised review articles, all of the considerations in this article will reduce this wide research scope to certain peculiarities when using XFEM, including its chosen modifications, in the above-mentioned analysis. Following this introductory Section 1, Section 2 provides a summary of the physical preparations required for the computational methods mentioned above. The summary of mathematical models will be covered in Section 3, with the details of XFEM-based methods; other numerical approaches are described in Section 4. In this regard, many relevant cases from the authors’ research experience are presented in Section 5. Next, Section 6 proposes new XFEM-motivated techniques, along with how they relate to previous approaches. The results are summarized in Section 7, along with suggested lines of research.

## 2. Physical Preliminaries

**Figure 1:**
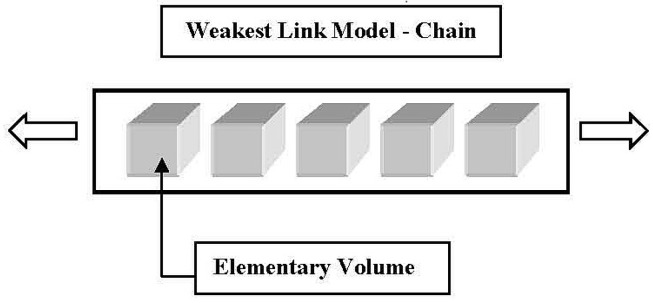
Local approach principle.

**Figure 2:**
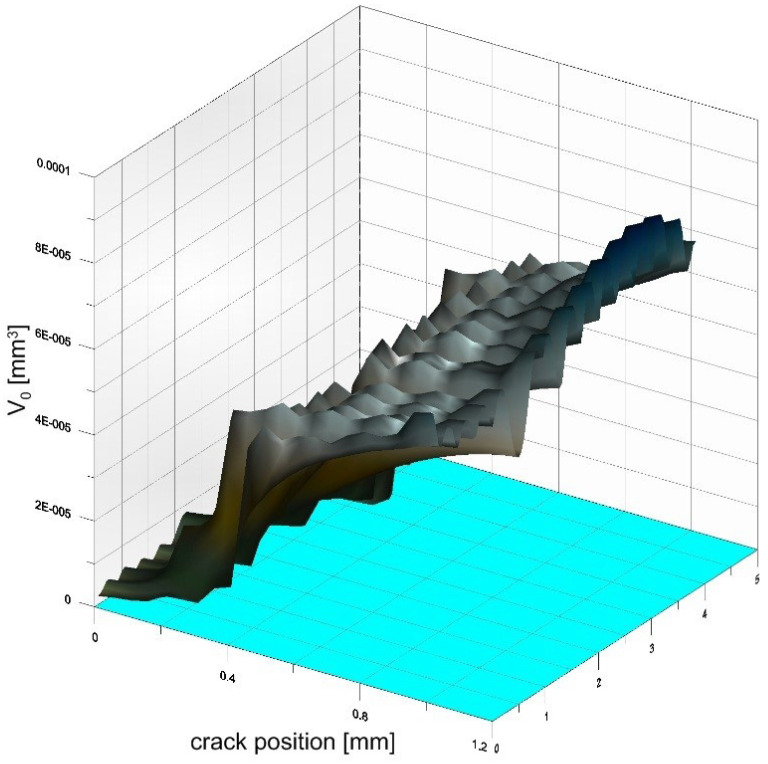
Crack-tip growth based on the size of the elementary volume $V_{0}$ [34].

Crack initiation and propagation occur progressively in brittle fracture. Most steels experience crack nucleation in brittle particles at grain boundaries (such as carbides) as a result of stress concentration brought on by the build-up of dislocations on these particles—or in the case of composites, at the material interface. This clarifies why local plastic deformation frequently occurs before fracture. The weakest-link concept (see Figure 1) is the basic principle of the local approach to fracture. It assumes that the failure of a body of material containing a large number of statistically independent volumes is caused by the failure of one of the reference volumes $V_{0}$ (see Figure 2), which has been identified as the volume of material related to the probability of finding a statistically significant number of microcracks. In finite element analysis, it is kept constant; in order to calculate the local stress independently of the finite element mesh used, the element size in this zone before the crack tip must be smaller than the size $V_{0}$.

**Figure 3:**
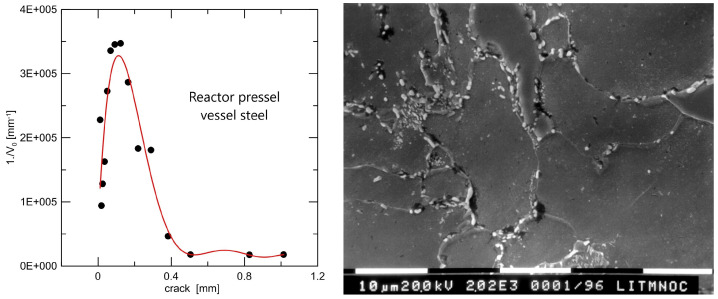
Example of varying size of the elementary volume $V_{0}$ ahead the crack tip and carbide particles observed on the grain boundaries [34,35].

Crack nuclei (CNs) that do not appear unstable during development eventually become blunt and stop taking part in the fracture initiation process. The plastic strain level determines the rate of CN production per unit volume ρ. It is significant because there may be a change of more than an order of magnitude in the local plastic strain prior to a crack or notch. Furthermore, ρ is significantly impacted by temperature [33]. From a mathematical perspective, the reference volume, $V_{0}=1/\rho$, which is recognised in the majority of traditional models, will not be constant. Temperature *T* and local plastic strain both affect its magnitude. An illustrative example of the variable size of the elementary volume $V_{0}$ for structural steel can be found in Figure 3.

A different approach must be chosen in the case of composite materials, especially fibre composites, where numerous studies have examined composite failure from a micromechanical perspective. Four fundamental mechanisms are usually involved in the failure process when a crack spreads in a composite material perpendicular to the direction of reinforced fibres: (i) matrix cracking, (ii) fibre/matrix debonding, (iii) fibre breaking, and (iv) fibre pull-out. Interfaces between the matrix and reinforcing fibres are crucial issues in the use of these materials [36]. The fracture toughness is one of the main important aspects of the interface, which is a highly rigorous area of fracture behaviour and strength. This contact determines the mechanical and fracture behaviour; it is essential for the transfer of stress between the reinforcement and the matrix. The common influence between the tangential and normal directions at the interface provides the separation. Compound materials are made up of two or more parts that complement one another with diverse qualities. The superior qualities of the other components balance out the deteriorating qualities of one. The microstructure’s morphological characteristics, including volume fraction, the size and spatial distribution of reinforcements, interfacial strength, and defect size, all affect how damage develops [37].

**Figure 4:**
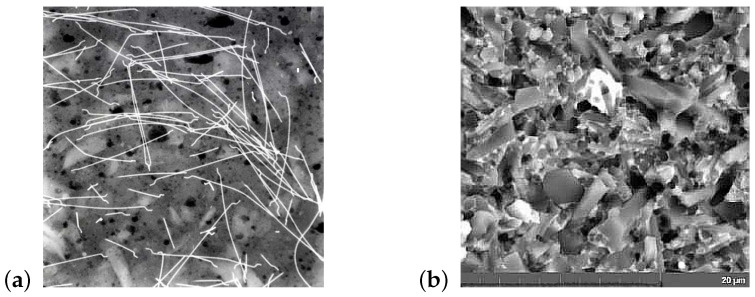
(**a**) Cementitious fibre composite reinforced by hook-end steel fibres of diameter 0.75 mm and length 35 mm (left-hand-side photo), [31]. (**b**) Surface of the ${\mathrm{Si}}_{3}N_{4}$ compound, 20 μm scale (right-hand-side photo), [35].

The sizing applied to the glass fibres during manufacturing controls the interfacial properties of glass fibre composites; these characteristics change when the size is altered. This has an impact on mechanical characteristics, e.g., fracture toughness and strength. In composite design, crack initiation is characterised by strength, whereas crack growth and damage progression are determined by fracture toughness. When a crack grows along the path of the fibre during mode I cracking, bridging takes place [38]. This failure pattern is crucial for fibre composite delamination and crack splitting around notches and holes. Failure is not just determined by cracking; the fibre bridging zone must be modelled as a separate mechanism (see Figure 4a).

A relatively new group of materials that can be modelled using techniques such as fibre composites is ceramics. Here, the influence of the fibre is actually replaced by the influence and behaviour of individual grains. For contemporary bearing applications, ceramics like silicon nitride (${\mathrm{Si}}_{3}N_{4}$) are recognised as the top option. Nevertheless, elements are exposed to significant cyclic contact stresses during service, in addition to harsh working circumstances such as high temperatures, corrosive environments, or rolling [31]. By appropriately modifying the microstructure, which is predicated on an understanding of the operating conditions, the qualities of ceramics can be improved. Specifically, the impact of modifying the grain structure on silicon nitride’s fracture toughness is illustrated in Figure 4b. This provides examples of how border phase modification and grain bridging affect toughness and strength.

Clearly, the above-mentioned considerations, including the updates of geometrical configurations, must become a part of an overall physical formulation. Such a formulation comes from the conservation principles of classical thermodynamics [39], namely, from conservation of energy, mass, and (linear and angular) momentum, coming from the so-called first law of thermodynamics. These principles, formulated mathematically as boundary and initial value problems for partial differential equations (or their systems in general), must be supplied by appropriate constitutive relations, compatible with the second law of thermodynamics (with respect to the third, which is not needed here explicitly) for reasonable engineering applications, referred also as “laws”, e.g., in special cases, (1) the Fourier law for thermal conduction and energy conservation, (2) the Fick law for contaminant diffusion through a porous medium and mass conservation, or (3) the Hooke law for elastic deformation of a body under usual mechanical loads and momentum conservation, which can be upgraded to the Kelvin or Maxwell laws for serial or parallel viscoelastic models, etc. In this article, we shall accent the case (iii), i.e., the proper considerations on time-dependent strain–stress relations, irreversible in general: at least an elastic, a viscous, a plastic, and some damage components will be required. We shall also respect the fact that classical differential (strong) formulations of such equations are replaced by their variational, weak, or still other integral formulations in most present-day computational strategies, including the XFEM techniques introduced above.

In the framework of crack tips, internal factors may be added to the equilibrium variables. This makes it possible to model a wider range of materials’ behaviours, such as various non-Newtonian fluids and materials with internal structures. For example, rheological models, fibre suspensions, and liquid crystals are taken into consideration. The purpose of rational thermodynamics is to handle continuum thermodynamics in an axiomatic manner. The second law of thermodynamics uses the idea of objectivity and material symmetry to limit constitutive functions. This may be taken into account in the exploitation of dissipation, in addition to the balancing equations and inequalities. Compared to the standard technique, this yields less restrictive conclusions on constitutive functions.

An additional group of problems is then related to the design of material characteristics and both experimental work and mathematical and computational tools directed to their identification [40,41], i.e., to the collaboration in the analysis of direct sensitivity and inverse problems. The discussion of inverse problems for generalized elastic continua will be divided into a few closely related areas at the intersection of experimental characterization and multiscale modelling. The majority of models with generalized elasticity have an excessive number of parameters, some of which are difficult to calibrate and have unclear engineering meanings. Such an excessive number of parameters results in ill-posedness, that is, a lack of robustness and uniqueness in the inverse methods; even small changes to the input data might cause an unstable identification response. These models have generalized, non-standard external actions that could be difficult to perform and monitor during real experiments. Investigating alternative formulations that significantly reduce the number of factors and produce analytical and numerical micro–macro are therefore imperative.

## 3. Mathematical Modelling

**Figure 5:**
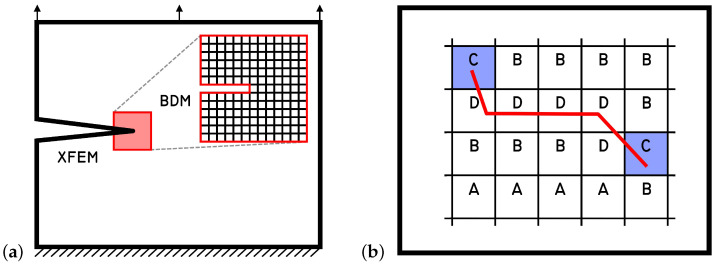
(**a**) Principles of multiscale analysis of a crack; the enrichment element is modified by BDM. (**b**) A typical FEM mesh with XFEM elements (A represents the standard elements, B partially enriched elements, C tip enriched elements, and D split enriched elements. Red line refers to a moving crack).

Reliable modelling of the development of strains and stresses in the above-mentioned materials and composites under mechanical, thermal, and other loads, including the danger of their damage, needs different formulations at the micro-, meso-, macro-, and other scales, typically along with some reasonable bridging of related results. An extensive overview of such multiscale approaches [42] comes from ab initio calculations up to macroscopic modelling of material specimens and whole structures. For illustration, simplified schemes for two-level modelling of crack propagation using XFEM and the bridging domain method (BDM) [43,44] are shown in Figure 5; more detailed comments on both XFEM and BDM will be presented in Section 4. From this rather wide research area, we shall pay particular attention to macroscopic modelling informed by certain mesostructural data. However, the physical, mathematical, and computational resources of recent engineering studies of this type [45,46,47,48,49,50,51] are not unified. Parallel development of mathematical scale-bridging convergence approaches seems to have its own rules and history, where only certain motivations stemming from engineering problems can be detected. In particular, in phase-field models working with some functional describing a distributed brittle fracture, a crucial issue is whether such a functional converges to that related to discrete fracture when a length scale introduced in the distribution function goes to zero.

The mathematical theory of homogenization of material characteristics, accompanying approaches aimed at scaling, is still far from being unified. Discussion on its history, classical results, and new achievements could generate a rather long specialised review article. Table 1 mentions only selected methods and algorithms with successful applications in engineering fracture mechanics or with great potential for such applications in the next several years.

Clearly, each of these table records must be seen as motivation for more detailed analysis. Specifically, the first one [52] covers several approaches, such as periodic two-scale convergence (between strong and weak convergence in standard functional analysis, Chap. 9) or certain of its non-periodic extensions (*G*-convergence and *H*-convergence, Chap. 11). Nevertheless, such mathematical results are not available (at least directly) in studies like [66], combining the weakest-link model in Figure 1 with microstructural randomness. A reasonable compromise can be seen in the concept of representative volume elements at particular scales [67], supplied with an appropriate scale-bridging optimization technique [68]. This can be coupled, e.g., with image processing [69] or with a stochastic technique supported by machine learning [70].

Nevertheless, most engineering studies still avoid deeper mathematical analysis entirely, preferring experimental validation of their results to formal existence, convergence, and other verification proofs. For various special classes of materials and structures [71,72,73,74,75], related XFEM (or similar) formulations are combined with stochastic modelling. However, some interesting results exceed such a simple characterisation, such as shape sensitivity analysis considering the uncertainty in crack geometry via the scaled boundary FEM [76], or probabilistic predictive modelling of curvature-crack spacing [77]. Recent studies have also tried to combine their numerical analysis of XFEM-type with selected soft computing techniques such as machine learning [78], or with artificial neural networks and Monte Carlo simulations [79]. Still other statistical approaches, such as Sobol sensitivity analysis [80] or exploitation of the theory of stochastic differential equations [81], are typical for simplified damage prediction under cyclic loading, which is beyond the scope of this article.

To support the comparison of various mathematical and computational approaches, we shall introduce a sufficiently simple model problem, direct and deterministic, related to case 3 in Section 2. From the physical point of view, we shall consider a non-polar deformable body; thus, the formulation of linear momentum will be sufficient. From the geometric point of view, we shall work with the usual small strain linearisation, with all displacement components related to their zero initial state.

Let a deformable body occupy some domain Ω in the 3-dimensional Euclidean space $R^{3}$ on a finite time interval I=[0,T]; notice the possibility of a non-trivial limit passage T→∞. A Lipschitz boundary ∂Ω of Ω, consisting of two disjoint subsets Γ and Θ, is considered for simplicity. A domain Ω is supplied by the Cartesian coordinate system $x=(x_{1},x_{2},x_{3})$; *t* denotes a time from I. Dot symbols will be presented briefly instead of ∂/∂t, whereas $\phi_{i,j}\left(x\right)$ will mean $\partial \phi_{i}\left(x\right)/\partial x_{j}$ for some functions ϕ defined on Ω, ∂Ω, etc., and appropriate indices i,j∈{1,2,3}, together with matrices ε(ϕ) compounded from $\varepsilon_{ij}\left(\phi \right)=\left(\phi_{i,j}+\phi_{j,i}\right)/2$, interpreted as small strain matrices in computational mechanics for displacements $u\left(x,t\right)=(u_{1}\left(x,t\right),u_{2}\left(x,t\right),u_{3}\left(x,t\right))$ with values in $R^{3}$ related to the reference configuration u(x,0)=0 for any x∈Ω. If needed, such a reference configuration can be redefined for an appropriate t>0, even adaptively during the computation, with the aim of suppressing the principal disadvantage of such a simple geometric linearization, but this will be omitted here.

In addition to a real displacement vector u(x,t), for a weak integral formulation of our model problem, i.e., that working with the Galerkin technique in Section 1, we shall also need a virtual (test) displacement w(x), independent of *t*. It is useful to introduce the space of such virtual displacements and other (abstract) function spaces needed in our considerations using the notion of Lebesgue, Sobolev, and Bochner–Sobolev spaces, along with the corresponding lemmas and theorems from [82,83], as the main arguments for formal existence and convergence proofs [84]. Since Θ will refer to a supported part of the boundary ∂Ω in our model problem, it is reasonable to assume w(x)=(0,0,0) for any x∈Θ in the definition of such a space V, in order to also force u(x,t)=(0,0,0) for an arbitrary *t*, for some *u* mapping I to V. For the brevity of notation, for appropriate vector- or matrix-valued functions ϕ and ψ, we shall also write (i) (ϕ,ψ) instead of a sum of Lebesgue triple integrals $\phi_{i}\left(x\right)\psi_{i}\left(x\right)$ over x∈Ω, where i∈{1,2,3}; (ii) 〈ϕ,ψ〉 instead of a sum of Hausdorff surface integrals $\phi_{i}\left(x\right)\psi_{i}\left(x\right)$ over x∈Γ, where i∈{1,2,3}; and (iii) ((ϕ,ψ)) instead of a sum of Lebesgue triple integrals $\phi_{ij}\left(x\right)\psi_{ij}\left(x\right)$ over x∈Ω, where i,j∈{1,2,3}.

In such a notation, the equation of motion, relying on the conservation of linear momentum, reads

(1) $$\left(w,\rho \ddot{u}\right)+\left(\;\left(\varepsilon \left(w\right),\sigma \right)\;\right)=\left(w,f\right)+\langle w,g\rangle$$

for any w∈V; here, $f\left(x,t\right)=(f_{1}\left(x,t\right),f_{2}\left(x,t\right),f_{3}\left(x,t\right))$ denote the volume loads prescribed for x∈Ω, and t∈I, $g\left(x,t\right)=(g_{1}\left(x,t\right),g_{2}\left(x,t\right),g_{3}\left(x,t\right))$ represent the surface loads prescribed for x∈Γ and t∈I, whereas the still undefined symbol σ(x,t) refers to some symmetric matrix for x∈Ω and t∈I, whose relation to ε(u) must be analysed carefully. This is connected with the formulation of some constitutive equations, whose positive characteristics will be considered as time-independent; however, they are still allowed to depend on x∈Ω. Another such positive material characteristic is the material density ρ, occurring in the first left-hand-side additive term of (1).

All of the following considerations depend on the accepted material model. We shall refer, step by step, to the following particular cases here:

(1) Simple linear problem: pure elasticity.
(2) Still linear problem: viscoelasticity.
(3) The first type of nonlinear generalization: elastoplasticity.
(4) The second type of nonlinear generalization: microscopic damage.
(5) The third type of nonlinear generalization: macroscopic cracking.

Ad (1): The simplest relation between σ and ε(u) is the linear elastic one, well known as the Hooke law, whose general form is σ=Cε(u), where the values of C refer to the symmetric 4-order tensor. Since $\sigma_{ij}=\sigma_{ji}$ and $\varepsilon_{ij}\left(u\right)=\varepsilon_{ji}\left(u\right)$ for any x∈Ω and t∈I, we have only six different components of σ and ε(u), generating 6×6=36 needed components of C, i.e., 6×5/2+6=21 different ones with respect to the symmetry of C, understood as $C_{ijkl}=C_{jikl}=C_{ijlk}=C_{klij}$ for any i,j,k,l∈{1,2,3}. Such a large set of material characteristics is rarely implemented: recall that, in the isotropic case, all components of C can be derived from one pair of Lamé constants only, or alternatively from the Young modulus and the Poisson ratio. However, we can always insert σ=Cε(u) into (1) formally; the assumption of positive definiteness of C reads $\sum_{i,j,k,l\in \{1,2,3\}}C_{ijkl}a_{ij}a_{kl}\geq C_{*}\sum_{i,j\in \{1,2,3\}}a_{ij}a_{ij}$ for some positive constant $C_{*}$ and arbitrary elements $a_{ij}$, $a_{kl}$ of a symmetric matrix *a*. Unfortunately, in such a case, (1) represents a physically closed system, non-communicating with the environment, which is far from reality in most engineering applications; thus, a credible incorporation of energy dissipation is required.

**Figure 6:**
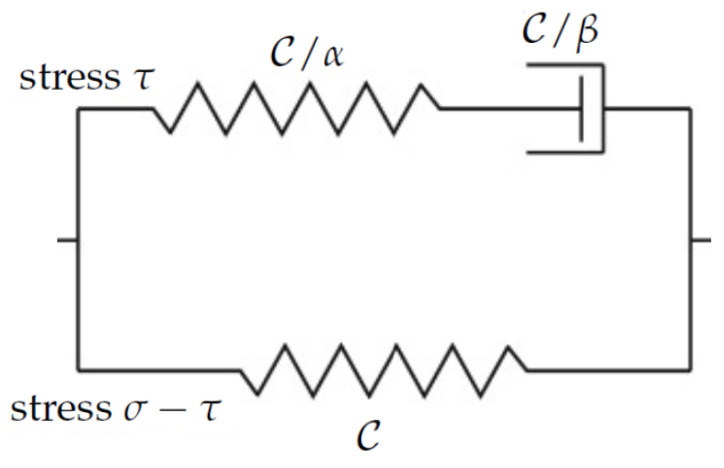
Engineering scheme of the viscoelastic SLS model.

Ad (2): This can be done using some viscoelastic model – but neither the linear serial Kelvin–Voigt one, nor the linear parallel Maxwell one, occurring in classical studies – as a basis for possible generalizations [85]. As a certain reasonable compromise, the so-called Standard Linear Solid (SLS) model will be utilized here, with scalar positive factors α and β for simplicity; its modification for more-component chains consisting of elastic and viscous components is straightforward [86]. The engineering scheme in Figure 6 can be expressed as σ−τ=Cε(u), $\alpha \tau =C\varepsilon (\dot{u}-\dot{\tilde{u}})$ and $\beta \tau =C\varepsilon (\tilde{u})$ with an a priori unknown displacement part $\tilde{u}$.

A very similar scheme is exploited in [87]; unlike this, the introduction of factors α and β in Figure 6 supports the discussion of classical limit cases below.

Since $\tilde{u}$ can be eliminated from the last two formulae easily, we are able to write

(2) $$\left(\;\left(\varsigma ,\alpha \dot{\tau }\right)\;\right)+\left(\;\left(\varsigma ,\beta \tau \right)\;\right)=\left(\;\left(\varsigma ,C\varepsilon \left(\dot{u}\right)\right)\;\right)$$

for a test function ς from a space H of functions introduced on Ω with values in $R^{3}\times R^{3}$, in addition to w∈V, and to modify (1) as follows:

(3) $$\left(w,\rho \dot{v}\right)+\left(\;\left(\varepsilon \left(w\right),\tau \right)\;\right)+\left(\;\left(\varepsilon \left(w\right),C\varepsilon \left(u\right)\right)\;\right)=\left(w,f\right)+\langle w,g\rangle$$

where, avoiding the second-order derivative in (1) with help of the displacement rates $v=\dot{u}$ formally, we have

(4) $$\left(w,\rho \dot{u}\right)=\left(w,\rho v\right)\;.$$

As three initial conditions, we shall consider u(x,0)=(0,0,0), $v\left(x,0\right)=\dot{u}\left(x,0\right)=\left(0,0,0\right)$, and τ(x,0) as the zero-valued square matrix of order 3 for all x∈Ω for simplicity. Note that, for the simplest viscoelastic models, a separate integral equation like (2) may be not necessary. To demonstrate this, let us consider γC in the lower branch of Figure 6 instead of C formally, with γ being a non-negative real constant, and admit α≥0 on Ω instead of α>0 in the upper branch, i.e., the elastic model component C/α vanishes with $u=\tilde{u}$ on such a hypothetical part of Ω where α=0. As the first special case, let us set γ=1 and α=0 on Ω everywhere. We receive a simple algebraic constitutive equation, joining the second additive term to the Hooke one only, i.e., $\sigma =C\varepsilon \left(u\right)+C\varepsilon \left(\dot{u}\right)/\beta$, corresponding to the Kelvin–Voigt model. As the second special case, let us set γ=0 and α>0 on Ω everywhere. Thanks to σ=τ, we obtain a linear differential constitutive equation $\alpha \dot{\sigma }+\beta \sigma =C\varepsilon \left(\dot{u}\right)$, corresponding to the Maxwell model. which can be solved analytically for an unknown σ if necessary.

Ad (3): All equations of (2)–(4) are still linear but formulated in infinite-dimensional function spaces for x∈Ω (including its traces on ∂Ω) and t∈I. Their analytical or semi-analytical solutions, suitable for computational evaluations, are not available or (at least) very complicated, especially with the expectable perspective of their nonlinear generalizations. Specifically, (3) is unable to respect the quite different behaviour of numerous materials and composites under tension and compression, as shown by the cementitious matrix in Figure 4a. However, this can be handled by adding some plastic part η(σ) to ε(u) where ${\dot{\eta }}_{ij}\left(\sigma \right)=\kappa \;\partial G\left(\sigma \right)\;/\partial \sigma_{ij}$ for particular i,j∈{1,2,3} using an appropriate plastic potential G(σ) and a plastic flow parameter κ; this basic concept of associated plastic flow can be generalized in several directions [88]. Consequently, (3) obtains one more right-hand-side additive term ((ε(w),Cη(σ))). A corresponding physical process is active only if the yield criterion F(σ,η(σ))=0 is satisfied by σ; a parameter κ is contained there. Both eventual loading and unloading can be characterized by three Karush–Kuhn–Tucker relations (equations and inequalities) from the classical optimization theory [89]. In general, such a form of incorporation of η(σ) is prepared for an iterative evaluation with a special choice of *F* and *G*. In particular, for the above-mentioned case from Figure 4a, this can be done using the Rankine-type criterion for tensile strength together with the Hill-type criterion for compressive strength [90].

Ad (4): Another type of nonlinearities comes from an attempt to include the effect of microfracture into our model problem, thanks to the stiffness reduction driven by a certain damage factor D(σ) on Ω with values between 0 and 1. Specifically, C is replaced by (1−D(σ))C, where D(σ) comes from a rather complicated algorithm with several additional material parameters and some nonlocal regularization steps [19,20], needed also in formal mathematical existence and convergence proofs [84], relying on [83] (two proofs of the same regularization result on operators in Lebesgue spaces of square integrable functions in Section 2.2). Recall that D(σ)=1 means the total loss of stiffness on some part of Ω locally; thus, we cannot expect realistic computational results in such a simplified model formulation, which is also reflected by the difficulties in mathematical proofs. In general, we have to implement ((ε(w),(1−D(σ))Cε(u))) instead of ((ε(w),Cε(u))) in (3). Applying the same approach to (2) is more delicate because of the presence of a time derivative: in this case, it may be better, using the simplified notation [ϕ] for an integral of a function $\phi (\tilde{t})$ over $\tilde{t}$ from 0 to *t*, to rewrite (2), considered for $\tilde{t}\in I$ instead of *t* formally, as

(5) ((ς,ατ))+[((ς,βτ))]=((ς,Cε(u)))

and to apply ((ς,(1−D(σ))Cε(u))) instead of ((ς,Cε(u))) to (5). Note that stress invariants derived from σ are typically taken instead of all particular components of σ, in order to guarantee the objectivity of such formulations, i.e., their independence of the choice of (local or global) coordinates.

Ad (5): However, we still have a model problem working with just one deformable body in Ω, whereas contact and potential impact of several deformable bodies may be required [91]. Fortunately, the same formulation can be repeated for a union of a finite number of deformable bodies, whose mutual contacts form a union of surfaces Λ. This seems to be similar to the introduction of surface loads *g* on Ω, but such surface tractions T on Λ may depend on displacement jumps δu of *u* from both sides adjacent to particular subsets of Λ, whose signs (unlike those of *g*) depend on the artificially defined orientation of “outward” normal directions to Λ, and on some additional material parameters. Consequently, with displacement jumps δw also applied to *w*, we have to add some 〈|δw,T(δu)|〉 to 〈w,g〉 in (3), where 〈|.,.|〉 means the same on Λ as 〈.,.〉 on Γ. This approach is also applicable to further potential parts of Λ, characterized as predefined or adaptively initiated cracks, with some expectable technical difficulties in the second case, such as the danger of loss of Lipschitz continuity in the boundaries of some domains, making standard embedding, trace, and other theorems in Sobolev spaces from [82,83] for the analysis of partial differential equations weaker and more complicated [92,93]; T(δu) can then express a traction separation law in a cohesive mode. Recall that the general mathematical description of the initiation and development of interacting cracks as cohesive interfaces relies on the nonlocal evaluation of *J*-integrals or comparable characteristics [94,95].

The cohesive approach allows for solving a number of practical applications for various types of materials and loads using a minimal number of parameters. The special law of material separation is mainly given by the cohesive stress $T_{0}$ and the cohesive energy $\Gamma_{0}$. Appropriate experimental methods have been developed to determine these material parameters. In a first approximation, the cohesive energy is determined by the critical value *J* of the integral [31]. One of the key problems in the application of the cohesive model is the selection of material laws within the cohesive region that describe the behaviour of the material in the local region in front of the crack front. The shape of this law may be smooth (such as an exponential) or may include fibre-pulling and bridging effects [21,24,96]. As another instructive example, [87] demonstrates the comparison of viscoelastic, elastoplastic, and elastic vs. smeared damage approaches for the experimentally supported case of the dynamic impact of a steel punch into a concrete beam, based on the dissipative energy evaluation.

## 4. Computational Implementation

Let us come back to an original linear model problem ((2)–(4)). There are two basic approaches to its theoretical and numerical analysis [82]: the Rothe method (Part 8.2) and the Faedo–Galerkin method (Part 8.4). The former starts with the time discretisation of I, which makes it possible to covert an original weakly formulated system of parabolic Equations (2) (or (5) alternatively), (3), and (4) to a sequence of elliptic equations, still in infinite-dimensional function spaces H and V. The existence of a solution can be studied using the convergence properties of at least two types of Rothe sequences: linear Lagrange splines for the approximation time derivatives, and simple functions for all other cases. The design of practical computational algorithms needs an additional approximation of H and V, for our model problem is based on XFEM or similar techniques for the discretisation on Ω and all parts of ∂Ω, typically resulting in a repeated analysis of large, sparse systems of linear algebraic equations; such a linearity must be forced by some iterative procedure in the sense of all considerations following (4) in Section 3.

The second approach starts only with the discretisation of Ω, which converts an original system of parabolic equations to a large, sparse system of ordinary differential equations, which could be solved—at least for linear problems, theoretically—using the generalized eigenvectors and eigenvalues of such a system. From the computational point of view, this requires a robust and effective evaluation of such eigenvectors and eigenvalues, which is rarely available, along with the need for iterative procedures for nonlinear generalizations. A usual remedy relies on additional time discretisation; this leads to a repeated analysis of large, sparse systems of linear algebraic equations, similar to the first approach. For a linear model problem, both approaches can be seen as equivalent, which may be not true for special types of additional nonlinearities. However, we prefer the first approach in the following sketch of computational implementation.

Let us come back to the system (2)–(4). We shall consider a finite number of discrete times $t_{s}=sh$ on I, with mh=T, s∈{1,…,m}, and approximate unknown $\tau (.,t_{s})$, $u(.,t_{s})$, and $v(.,t_{s})$ (where dots substitute *x*, needed in integrals) by some $\tau_{s}\in H$, $u_{s}\in V$, and $v_{s}\in V$, with the aim (for theoretical analysis) m→∞. A priori known time-dependent functions, such as $f(.,t_{s})$ and $g(.,t_{s})$, can also be approximated by a certain $f_{s}$ and $g_{s}$, e. g., using the Clément quasi-interpolation [82], based on evaluation of mean values on subintervals [(s−1)h,sh]. Clearly, all components of $\tau_{0}$, $u_{0}$, and $v_{0}$ for $t=t_{0}=0$ are formally zero-valued, thanks to our set of homogeneous initial conditions. Thus, we obtain

(6) $$\begin{array}{ccc} \left(\;\left(\varsigma ,\alpha \left(\tau_{s}-\tau_{s-1}\right)\right)\;\right)+h\left(\;\left(\varsigma ,\beta \tau_{s}\right)\;\right)\;\;\;\; & = & \;\;\;\;(\;\left(\varsigma ,C\varepsilon \left(u_{s}-u_{s-1}\right)\right)\;)\;, \end{array}$$

(7) $$\begin{array}{ccc} \;\;\;\;\;\;\;\;\;\;\;\;\;\;\left(w,\rho \left(v_{s}-v_{s-1}\right)\right)+h\left(\;\left(\varepsilon \left(w\right),\tau_{s}\right)\;\right)+h\left(\;\left(\varepsilon \left(w\right),C\varepsilon \left(u_{s}\right)\right)\;\right)\;\;\;\; & = & \;\;\;\;h\left(w,f_{s}\right)+h\langle w,g_{s}\rangle \;, \end{array}$$

(8) $$\begin{array}{ccc} (w,\rho \left(u_{s}-u_{s-1}\right))\;\;\;\; & = & \;\;\;\;h(w,\rho v_{s})\;, \end{array}$$

step by step for s∈{1,…m} and unkonwn $\tau_{s}$, $u_{s}$, and $v_{s}$. An alternative of (6), motivated by (5), reads

(9) $$\left(\;\left(\varsigma ,\alpha \tau_{s}\right)\;\right)+h\left(\;\left(\varsigma ,\beta \left(\tau_{1}+\ldots \tau_{s}\right)\right)\;\right)=\left(\;\left(\varsigma ,C\varepsilon \left(u_{s}\right)\right)\;\right)\;.$$

The effect of numerical integration in practical calculations is not emphasized here explicitly; thus, $f_{s}$, $g_{s}$, and all time-independent material characteristics in (6)–(9) stay unchanged.

A subsequent discretisation in $R^{3}$ can adopt the formal notation of (2)–(4): ς∈H can be replaced by some $\varsigma^{d}$ from a finite-dimensional space $H^{d}$, approximating H and w∈V by some $w^{d}$ from a finite-dimensional space $V^{d}$, approximating V in a reasonable way for an appropriate length measure d in $R^{3}$ tending to zero. Consequently we are searching for $\tau_{s}^{d}\in H^{d}$, $u_{s}^{d}\in H^{d}$$u_{s}^{d}\in H^{d}$ satisfying

(10) $$\begin{array}{ccc} \left(\;\left(\varsigma^{d},\alpha \left(\tau_{s}^{d}-\tau_{s-1}^{d}\right)\right)\;\right)+h\left(\;\left(\varsigma^{d},\beta \tau_{s}^{d}\right)\;\right)\;\;\;\; & = & \;\;\;\;(\;\left(\varsigma^{d},C\varepsilon \left(u_{s}^{d}-u_{s-1}^{d}\right)\right)\;)\;, \end{array}$$

(11) $$\begin{array}{ccc} \;\;\;\;\;\;\;\;\;\;\;\;\;\;\left(w^{d},\rho \left(v_{s}^{d}-v_{s-1}^{d}\right)\right)+h\left(\;\left(\varepsilon \left(w^{d}\right),\tau_{s}^{d}\right)\;\right)+h\left(\;\left(\varepsilon \left(w^{d}\right),C\varepsilon \left(u_{s}^{d}\right)\right)\;\right)\;\;\;\; & = & \;\;\;\;h\left(w,f_{s}\right)+h\langle w,g_{s}\rangle \;, \end{array}$$

(12) $$\begin{array}{ccc} (w^{d},\rho \left(u_{s}^{d}-u_{s-1}^{d}\right))\;\;\;\; & = & \;\;\;\;h(w^{d},\rho v_{s}^{d})\;. \end{array}$$

An alternative of (10), exploiting (9), is

(13) $$\left(\;\left(\varsigma^{d},\alpha \tau_{s}^{d}\right)\;\right)+h\left(\;\left(\varsigma^{d},\beta \left(\tau_{1}^{d}+\ldots \tau_{s}^{d}\right)\right)\;\right)=\left(\;\left(\varsigma^{d},C\varepsilon \left(u_{s}^{d}\right)\right)\;\right)\;.$$

The seemingly sufficient choice of $V^{d}$ and $H^{d}$ based on linear splines on Ω, extended to Γ and Θ, as well as advanced adaptive FEM strategies [97,98], will need revisions due to the rather complicated structure of Λ and to the exploitation of XFEM.

Note that (6)–(8) exploit the classical implicit (backward) Euler scheme, to avoid difficulties with conditional stability [99]. Certain semi-implicit approaches will also be outlined for nonlinear generalizations. Development of more advanced time integration schemes, e. g., of the Runge–Kutta type, can be based on the algebraic analysis of *B*-series [100].

Since all nonlinear contributions to (2)–(4), discussed in Section 3 under (5), occur rarely together in particular computational studies, we shall outline them separately. The first one brings a new term $\left(\;\left(\varepsilon \left(w\right),C\eta \left(\sigma_{s*}\right)\right)\;\right)$ to the right-hand side of (7) and, consequently, a new term $\left(\;\left(\varepsilon \left(w^{d}\right),C\eta \left(\sigma_{s*}^{d}\right)\right)\;\right)$ to the right-hand side of (11); a lower index $\phi_{s*}$ (here and later) instead of $\phi_{s}$ for a function ϕ (here and later) means a reasonable estimate of $\phi_{s}$: in the first attempt, perhaps $\phi_{s*}=\phi_{s-1}$. Thus, (11) stays linear apart from the fact that some iterative improvements of $\sigma_{s*}^{d}$ (whose evaluation is rather expensive), close to a proper $\sigma_{s}^{d}$, will be needed.

The second contribution brings a modified term $\left(\;\left(\varepsilon \left(w\right),\left(1-D\left(\sigma_{s*}\right)\right)\;C\varepsilon \left(u_{s}\right)\right)\;\right)$ to the left-hand side of (7) and a modified term $\left(\;\left(\varsigma ,\left(1-D\left(\sigma_{s*}\right)\right)\;C\varepsilon \left(u_{s}\right)\right)\;\right)$ to the right-hand side of (9); consequently, a modified term $\left(\;\left(\varepsilon \left(w^{d}\right),\left(1-D\left(\sigma_{s*}^{d}\right)\right)\;C\varepsilon \left(u_{s}^{d}\right)\right)\;\right)$ is brought to the left-hand side of (11), and a modified term $\left(\;\left(\varsigma^{d},\left(1-D\left(\sigma_{s*}^{d}\right)\right)\;C\varepsilon \left(u_{s}^{d}\right)\right)\;\right)$ is brought to the right-hand side of (13). The arguments on the need for iterative improvements of $\sigma_{s*}^{d}$ can be repeated from the preceding case.

The third contribution brings a new term $\langle \;|\delta w,T\left(\delta u_{s*}\right)|\;\rangle$ to the right-hand side of (7) and, consequently, a new term $\langle \;|\delta w^{d},T\left(\delta u_{s*}^{d}\right)|\;\rangle$ to the right-hand side of (11). This demonstrates the need for iterative improvements of $u_{s*}^{d}$, following that of $\sigma_{s}^{d}$. Another consequence of this contribution is the necessity of updates to Λ, including an adaptive (potentially multiple) crack system, which generates substantial requirements for the XFEM-based approximations.

Multiscale representation of materials—covering hierarchical structures from electron density to the continuum level, such as the design of a unified data-driven framework integrating information across scales to address deformation and fracture phenomena—is a challenge of recent fracture mechanics: from quantum mechanics working with electrons and other elementary particles step by step, to molecular dynamics working with atoms, to mesomechanics handling a certain quasi-continuum, up to continuum mechanics used in engineering practice [101]. Consequently, 25 critical and unresolved questions have been distinguished [102], corresponding to frontiers of physical and mathematical theory, materials analysis, and engineering applications, exceeding the scope of this article. We shall illustrate this progressive research area based solely on the practical design of (11), with evident consequences for (12) and (10) or (13), open to the class of nonlinear generalizations outlined above, i.e., for the third contribution with discrete crack discontinuities here.

Modelling of brittle, quasi-brittle, and ductile fractures can typically exploit the level-set approach [103]. Two level-set functions, originally designed for linear elastic fracture mechanics, can be used: (i) a jump function to capture displacement jump across the crack facets, and (ii) an asymptotic branch function that spans the asymptotic crack-tip field. Such an enrichment approximation takes the form

(14) $$u_{s}^{d}\left(x\right)=\sum_{I\in S^{e}}N_{I}\left(x\right)u_{sI}^{d}+\;\sum_{J\in S^{c}}N_{J}\left(x\right)\left(\Psi \left(x\right)-\Psi \left(x_{J}\right)\right)a_{sJ}^{d}+\;\sum_{K\in S^{f}}N_{K}\left(x\right)\;\sum_{A=1}^{n}\Theta_{A}\left(r,x\right)b_{sKA}^{d}\;.$$

Here, a positive d and a time step index s∈{1,…,m} comes from (11). $S^{e}$ refers to the nodes from standard FEM, $S^{c}$ to the nodes where elements are catted via proper criterion, and $S^{f}$ nodes where the crack tip is supplied with a fixed distance *r* independent of the mesh size characterized by d; Ψ(x) and $\Theta_{A}\left(r,x\right)$ are appropriate enrichment functions, where A∈{1,…n} for some finite *n*, frequently reformulated in spherical coordinates in the case of $\Theta_{A}$. In particular, for isotropic materials, such n=4 functions replacing $\Theta_{A}\left(r,x\right)$ could be $\Theta_{1}\left(r,\theta \right)\;=\;\sqrt{r}\sin\left(\theta /2\right)$, $\Theta_{2}\left(r,\theta \right)\;=\;\sqrt{r}\cos\left(\theta /2\right)$, $\Theta_{3}\left(r,\theta \right)\;=\;\sqrt{r}\sin\theta \sin\left(\theta /2\right)$, and $\Theta_{4}\left(r,\theta \right)\;=\;\sqrt{r}\sin\theta \cos\left(\theta /2\right)$ for a certain dimensionless angle θ.

For multiscale modelling, the bridging domain method, briefly mentioned in the first paragraph of Section 3, is often used. It can be classified as a domain decomposition coupling method (see Figure 5). The basic domain Ω is decomposed into two overlapping domains $\Omega_{C}$ and $\Omega_{S}$, where $\Omega_{C}$ corresponds to a subdomain where FEM, XFEM, or a similar computational technique is used, whereas an appropriate multiscale model is applied to $\Omega_{S}$. Especially for two scales, let the subdomains $\Omega_{C}$ and $\Omega_{S}$ overlap on a certain set $\Omega_{H}=\Omega_{C}\cap \Omega_{S}$. Then, it is natural to introduce a weight function ω on Ω as equal to zero on $\Omega_{C}\;\setminus \;\Omega_{S}$ (purely continuous modelling), one on $\Omega_{S}\;\setminus \;\Omega_{C}$ (purely lower-scale modelling), and some other values on $\Omega_{H}$ in a way that preserves the continuity of ω on Ω.

Inserting $w^{d}=u_{s}^{d}$ into (11) formally, we are able, with respect to (12) and (10) or (13), to introduce

(15) $$\begin{array}{ccc} W_{C}\left(\omega \right)\;\;\;\; & = & \;\;\;\;\frac{1}{2}\left(v_{s}^{d},\omega \rho v_{s}^{d}\right)+h\left(\;\left(\varepsilon \left(u_{s}^{d}\right),\omega \tau_{s}^{d}\right)\;\right)+h\left(\;\left(\omega \varepsilon \left(u_{s}^{d}\right),\omega C\varepsilon \left(u_{s}^{d}\right)\right)\;\right)\\ \;\;\;\; & - & \;\;\;\;h\left(u_{s}^{d},\omega f_{s}\right)-h\langle u_{s}^{d},\omega g_{s}\rangle \;. \end{array}$$

Recall that it is sufficient to calculate $W_{C}\left(\omega \right)$ by (15), with the help of (14), on $\Omega_{C}$ only. Suppose that some $W_{S}\left(1\;-\;\omega \right)$ as an analogy of $W_{C}\left(\omega \right)$ in (15) can be evaluated for a lower scale on $\Omega_{C}$, as from the technique of activation and deactivation of virtual atoms [104], with some ${\tilde{u}}_{s}^{d}$ replacing $u_{s}^{d}$ from (15). Clearly, the form of (15) is inspired by the evaluation of the total system energy approximated on Ω and on its boundary at a particular time $t_{s}$, whereas the specific form of $W_{S}$ may depend on the considered material type and on the incorporated scale level(s). However, we have

(16) $$W_{C}\left(\omega \right)+W_{S}\left(1\;-\;\omega \right)={\left(\lambda ,u_{s}^{d}-{\tilde{u}}_{s}^{d}\right)}_{H}$$

in general, where λ refers to a vector-valued Lagrange multiplier and ${\left(.\;,.\right)}_{H}$ forces the integration over $\Omega_{H}$ instead of Ω (where both $u_{s}^{d}$ and ${\tilde{u}}_{s}^{d}$ are available) only, i.e., $u_{s}^{d}\approx {\tilde{u}}_{s}^{d}$ in a reasonable energetic sense.

Note that concurrent and hierarchical frameworks are often considered as two basic computational approaches, capable of bridging different scales from atomic to continuum descriptions. For problems with a clear separation between scales, i.e., for the transparent evaluation of terms like $W_{S}\left(1\;-\;\omega \right)$ in (16), hierarchical modelling passes information sequentially from small to large scales. Concurrent modelling couples different scales dynamically, which may be essential in the analysis of localised phenomena where scales cannot be distinguished in a simple way. The physical consistency of such approaches relies on such principles as the Cauchy–Born rule [105] and the Hill–Mandel condition [106] for crystal lattices, including certain generalization beyond them [107,108].

Recall that (11) with (14) and similar equations—as a result of the discretisation of a model deterministic problem in $R^{3}$, evaluated in sequential time steps—do not include any uncertainty considerations explicitly. However, such considerations can be included using loads $f_{s}$ and $g_{s}$ with appropriate probabilistic distributions, validated by data from measurements and observations. The same may also be done with material parameters C, as well as with α and β in (10), ρ in (11), etc. Such an approach is open even to formulations with scale bridging. Consequently, numerical solutions of large, sparse systems of equations (nonlinear in nearly all practical cases) are needed; this requires extensive parallel simulations, i.e., those using a Monte Carlo-based approach [109].

## 5. Illustrative Example

**Figure 7:**
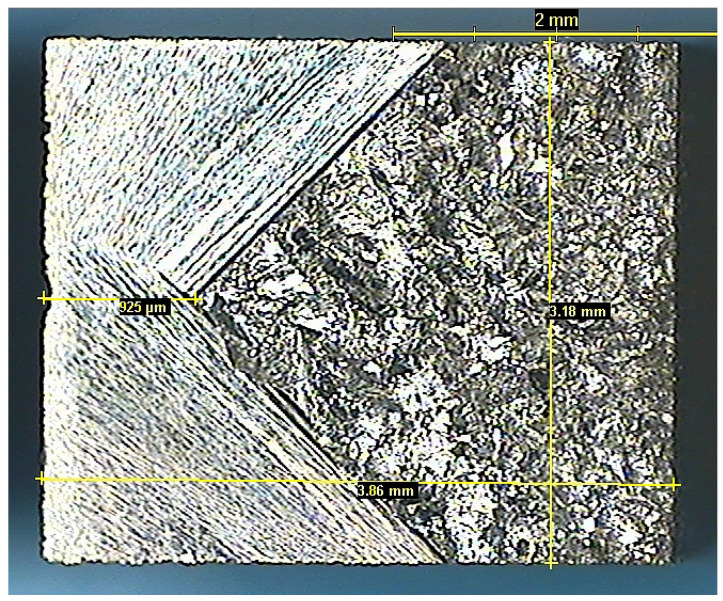
Sharp chevron notch; prediction quality depends on repeatability of geometry for a set of experiments [110].

As a simple example, the intermetallic alloy Ti−46Al−0.7Cr−0.1Si−7Nb−0.2Ni with a sharp notch was chosen as the model material. Because of their exceptional mechanical qualities and low density, particularly at high temperatures, intermetallics are regarded as one of the most prominent “composite” materials. Their primary application is in the aerospace sector, particularly in turbine parts that are subjected to high temperatures and have the power to entirely substitute nickel alloys. The crack propagation prediction was performed using XFEM; the material behaviour was described by a cohesive model with a shape similar to an exponential law. For the bending strength tests at three-point and four-point bending, beam-type test specimens with a cross-section of approximately 3.2 × 4.1 mm and a length of approximately 45 mm were used. To determine the fracture toughness, the method of test specimens with a chevron notch, which were cut with a diamond wheel, was used; the shape is evident from Figure 7. The fracture toughness *K* was determined from the maximum strength and depth of the chevron.

**Figure 8:**
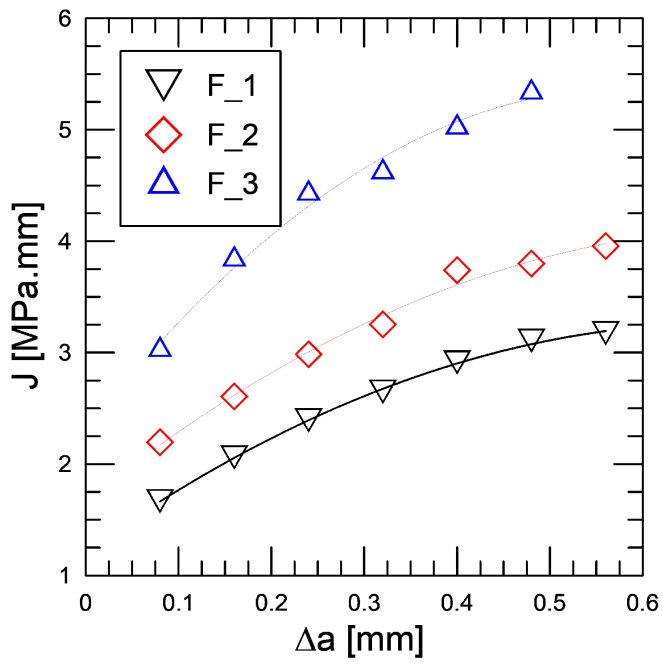
Crack growth–resistance curve J−Δa for fracture toughness values K=14 MPa $m^{1/2}$ (F 1), K=16 MPa $m^{1/2}$ (F 2), and K=20 MPa $m^{1/2}$ (F 3), with Δa understood as crack extension [112].

The system continuously calculated the value of the *J*-integral at the crack front, cf., the discussion in the second-last paragraph of Section 3. The length of the crack was determined by the size of the element generated in the model. In this particular case, the edge length of the element was 0.08 mm. Young’s modulus was E=175,000 MPa, and Poisson’s ratio was ν=0.33, all under the yield stress 300 MPa. The traction separation law was given by the cohesive stress 550 MPa, whereas the cohesive energy was computed as $K^{2}/\left(E\left(1-\nu \right)\right)$. This makes it possible to evaluate the needed material parameters for $T(\delta u_{s*})$ using an appropriate optimization algorithm [111]. The above-mentioned probabilistic procedure can be applied to the behaviour of this material under possible combined loading, which could lead to a lower number of necessary experimental data for verification. Figure 8 shows how the predicted values of the crack growth–resistance curve correspond to the experimental data.

It turns out that the influence of the size of the plastic zone on the crack propagation area, when considering heterogeneities, has a similar behaviour as when solving the issue of the accuracy of the applied traction separation law. This means that higher plasticity reduces the influence of heterogeneity in a certain way, which encourages the use of statistical methods.

## 6. Perspectives, Future Works, and Expected Trends in Development

We have paid particular attention to the classical thermomechanical arguments supported by computational techniques and the corresponding mathematical background. However, the extensive use of advanced structural materials and composites has stimulated the development of other approaches in the last several years, including their classification in review articles. Some of these are briefly summarised in Table 2.

Unlike these computational results, validated by experiments and observations in numerous cases, the formal verification of mathematical theory [128] addressed to strong, weak, very weak, etc., solvability and convergence properties of approximate solutions is still far from being complete, at least excluding sufficiently simple and cautiously chosen benchmark problems. Further progress will probably need nonlinear homogenization [129,130] applied to an advanced continuum model, such as that based on a relaxed micromorphic continuum [131] and/or on a thermodynamic framework represented by a general equation for non-equilibrium reversible–irreversible coupling (GENERIC) [132].

Also very inspiring is the review [133], which deals with current trends in research into methods for the numerical modelling of fibre-reinforced concrete failures and the exploration of new approaches such as machine learning or artificial intelligence, encompassing a whole range of approaches at different levels. Nevertheless, it is impossible to overlook deep learning techniques. Even as data analysis and neural networks are becoming increasingly sophisticated, most of the work done to date is not based on substantial volumes of scientific data from which predictive models can be built using mechanistic assumptions and physical principles that have been empirically confirmed. Conversely, in the majority of applications, the physical conservation principles (such as for mass, momentum, and energy) are developed using very general mathematical formulations, such as those using partial differential equations [134].

Further development of the above-mentioned techniques and procedures dedicated to the issues defined in the *Abstract* can be characterized and possibly divided into several areas:

- Extension of applications for multi-level modelling based on energy descriptions expressed by appropriate notions from functional analysis, especially for lower levels, where hierarchical description is closer to the physical view. This, of course, corresponds to numerical approaches associated with the stability and convergence of computational algorithms, the existence of solutions, and determination by physically justified transition regions.
- In the corresponding theoretical part, a mathematical analysis of the modified finite element method for static, quasi-static, viscous, or plastic deformation and dynamic analysis of loaded deformable bodies of new material models will be performed. This could even include models based on molecular statistics, where the lattice of atoms is modelled as a set of points connected by nonlinear springs. Another similar approach is molecular dynamics, where the inertial forces of individual atoms are added. This could take us to the level of ab initio calculations, which was not the aim of this article. This, of course, applies not only to the first level of the hierarchical structure. However, this does not solely apply to issues related to deformation problems or heat conduction and the like.
- When combining this class of problems, where a new surface could generally occur or a crack could form and propagate, methods that have a constant number of nodes are clearly promising, i.e., they correspond to the class and procedures referred to in the XFEM application as intrinsic methods. Then, there can also be modification of shape functions and the use of the least squares method. This, of course, brings us to the purely technical problems of XFEM design and possible numerical tricks.
- In the case of a single-level approach, high demands are placed on understanding the processes in the local area opposite the crack front or a critical, relatively small area. An analysis of new procedures based on the traction-separation laws was performed.
- Various types of composites, especially fibre composites, represent a typical class of problems, where similar models can be used to solve problems that differ significantly in terms of the size of the local area and overall geometry. Two extremes can be pointed out, for example, the behaviour of cement composites and fibre-reinforced ceramics.
- We cannot forget the types of analyses that combine standard FEM or its modified version XFEM with stochastic procedures. This can of course be done as a single-level or multi-level model. Multi-level modelling seems more promising, but it can be complicated in terms of the algorithm and the possible use of criteria from the field of fracture mechanics. The methods, in a first approximation, can be characterised by the introduction of additional potentials, e.g., harmonic or atomic, defined by various rules such as non-classical Cauchy–Born, etc. In the general part, the bridging domain method is indicated, which is very representative for this class of problems. The numerical representation of these additional potentials is standard, and the construction of the corresponding matrices is almost problem-free.
- This above-mentioned problem can be eliminated by using models based on the classical Monte Carlo method, or some variant or generalization thereof [135,136]. From a statistical point of view, the usual approach is not hierarchical, although we can start from knowledge of the microstructure and apply the result to a higher level. However, we observe the influence of the elementary representative volume, RVE. The statistical method then acts as a criterion for the formation and propagation of a crack. A typical example is models based on the growth of voids, characterized by a certain distribution. From a numerical point of view, however, the load and time steps are very small, and problems with the convergence of the problem may occur. A lower number of experiments can be replaced by the Monte Carlo method for determining distribution functions. In the case of direct interactions with XFEM, the enrichment function can have both static and dynamic properties. Theoretically, statistical properties could be included in the cohesion law.
- In the above perspectives, we somewhat neglect the development of the FEM and XFEM methods themselves and their modifications. In the literature, the challenges associated not only with fracture and loading conditions are discussed. There are precise criteria describing the formation and growth of cracks based on a statistical approach that can replace the pure use of fracture mechanics methods for more complicated materials or operating conditions.
- It is possible to move from statistical methods to artificial intelligence. However, it is necessary to keep in mind the size and quantity of experimental (or verification) data, which is usually financially demanding.

## 7. Conclusions

The rapid current development of (i) engineering, (ii) physical, (iii) mathematical, and (iv) computational approaches—whose common motivation and inspiration is the development of advanced structural materials, including the reliable prediction of their potential damage behaviour—has been reflected and traced in this article, supported by its rather long list of references. Thus, it is not easy to augur future research directions, as well as the interconnections between existing and developing results of types (i), (ii), (iii), and (iv). However, with a certain apprehensible risk, the following brief list of general expectations can be formulated:

Future work could involve the introduction of a more accurate distribution of fracture properties, including the nonlinear dependence of the distribution and the standard deviation of the fracture energy toughness on the crack length, through further experimentation and statistical analysis. The integration of microscopic analysis with the stochastic modelling framework, XFEM, could also be useful for more sophisticated studies based on multi-level models. This would be advantageous in the case of partial parts belonging to an order-of-magnitude-larger structure. The predictive model serves to estimate the service life and safety of a structural element of the entire technical structure. Advanced materials with complicated responses under stress, such as composites, piezoelectric materials, and functionally graded materials, have been easier to research because of XFEM. Since XFEM can simulate crack propagation without the need for remeshing, it is a crucial tool for forecasting how these materials will behave under different circumstances, which helps in the creation of more robust and effective materials for special purposes, as well as possibly reacting to the material’s response to events taking place in a lower hierarchical structure. The capacity of XFEM to precisely simulate micro-scale and nano-scale fracture behaviours will be crucial as the field of materials science advances toward the creation of smart materials and nanotechnology. Future technological innovations, such as flexible electronics and biocompatible materials – i.e., those interacting with a living organism, as required in medical devices within or touching the human body – depend on the creation of next-generation materials, which will be aided by this capability. Attention should also be paid to any simplified reliance on methods based on artificial intelligence, as we do not always have a sufficient number of experiments to describe the behaviour of the material in question.

The above issue is still at the forefront of interest for both the mathematical community and practical numerical modellers, and it is to these communities that this article is dedicated, in the hope of enabling them to quickly navigate the modelling and prediction of the material behaviour of structures.

## Figures and Tables

**Table 1:** A brief list of homogenization approaches.

Methods	Source(s)
Classical homogenization approaches	[52]
FEM approximation in homogenization	[53]
Application of [52,53] to cohesive surfaces in fracture mechanics	[54]
Application of [52,53] to phase-field fracture	[55,56]
Introduction to Γ-convergence	[57]
Γ-convergence and variational evolution in plasticity versus damage	[58]
Classical stochastic homogenization	[59]
Σ-convergence for both deterministic and stochastic homogenization	[60,61]
Algebraic theory of homogenization structures	[62]
Stochastic approaches relying on [60,61,62]	[63,64]
Concurrent stochastic approach relying on Young measures	[65]

**Table 2:** Some useful review articles related to local approaches to crack development in structural materials.

Topics	Source
Classical review of XFEM, cohesive cracks, and least squares	[113]
XFEM for micro- and macrocracking	[114]
Comparison of XFEM, cohesive zone model, and peridynamics	[115]
Elastoplastic variants of XFEM	[116]
Extensions to fluid–structure interaction	[117]
Applications to concrete	[118]
Aapplications to pipelines	[119]
Applications to biocomposites	[120]
Applications to fibre-reinforced composites	[121]
Aapplications to welded joints	[122]
Regularized fracture modelling	[123]
Discrete fracture networks for multiphysical processes	[124]
Phase-field techniques	[125]
Damage to composite materials	[126]
Computational materials engineering; application to automotive industry	[127]

## Data Availability

No new data were created or analyzed in this study. Data sharing is not applicable to this article.
